# Determinants of late-stage cervical cancer presentation in Ethiopia: a systematic review and meta-analysis

**DOI:** 10.1186/s12885-023-11728-y

**Published:** 2023-12-14

**Authors:** Amare Zewdie, Solomon Shitu, Natnael Kebede, Anteneh Gashaw, Habitu Birhan Eshetu, Tenagnework Eseyneh, Abebaw Wasie Kasahun

**Affiliations:** 1 Department of Public Health, College of Medicine and Health Science, Wolkite University, Wolkite, Ethiopia; 2 Department of Midwifery, College of Medicine and Health Science, Wolkite University, Wolkite, Ethiopia; 3 Department of Health Promotion, School of Public Health, College of Medicine and Health Sciences, Wollo University, Dessie, Ethiopia; 4 Department of Midwifery, College of Medicine and Health Science, Dilla University, Dilla, Ethiopia; 5 Department of Health Promotion and Health Behaviour, Institute of Public Health, College of Medicine and Health Sciences, University of Gondar, PO.Box.196, Gondar, Ethiopia; 6 Department of Public Health, College of Medicine and Health Sciences, Injibara University, Injibara, Ethiopia

**Keywords:** Cervical cancer, Delayed diagnosis, Late-stage cancer, Cancer diagnosis, Ethiopia

## Abstract

**Introduction:**

Behind breast, colorectal, and lung cancers, cervical cancer is the fourth most common cancer affecting females. Despite, it is a preventable form of cancer both the incidence and mortality figures reflect it as a major reproductive health problem. Late-stage cervical cancer diagnosis is associated with complicated clinical presentation which can result in short survival time and increased mortality. Several factors contribute to the late-stage presentation of cervical cancer patients. In Ethiopia nationally summarized evidence on the level and the factors contributing to late-stage cervical cancer diagnosis is scarce. Therefore, this systematic review and meta-analysis aimed to assess the pooled prevalence of late-stage cervical cancer diagnosis and its determinants in Ethiopia.

**Method:**

A systematic review and meta-analysis were conducted using PRISMA guidelines. Comprehensive literature was searched in PubMed, Embase, Google Scholar, and African Online Journal to retrieve eligible articles. A weighted inverse variance random effect model was used to estimate pooled prevalence. Cochrane Q-test and I^2^ statistics were computed to assess heterogeneity among studies. Funnel plot and Egger’s regression test were done to assess publication bias.

**Result:**

Overall, 726 articles were retrieved and finally 10 articles were included in this review. The pooled prevalence of late-stage cervical cancer diagnosis in Ethiopia was 60.45% (95%CI; 53.04%-67.85%). Poor awareness about cervical cancer and its treatment (AOR = 1.55, 95% CI: (1.03 – 2.33, longer delay to seek care (AOR = 1.02, 95% CI: (1.01 – 1.03)) and rural residence (AOR = 2.07, 95% CI:( 1.56 – 2.75)) were significantly associated to late-stage diagnosis.

**Conclusion:**

In Ethiopia, six in every ten cervical cancer cases are diagnosed at the late stage of the disease. Poor awareness about cervical cancer and its treatment, long patient delay to seek care, and rural residence were positively associated with late–stage diagnosis. Therefore intervention efforts should be made to improve public awareness about cervical cancer, minimize patient delay to seek care, and expand screening services specifically in the rural residing segment of the population to detect the disease early and improve survival.

**Supplementary Information:**

The online version contains supplementary material available at 10.1186/s12885-023-11728-y.

## Introduction

Behind breast, colorectal, and lung cancers, cervical cancer is the fourth most common cancer affecting females [1]. Globally, in 2020, there were an estimated 604,127 cases and about 341,831 deaths of cervical cancer [2]. In developing nations, cervical cancer comes in second place behind breast cancer in terms of incidence and death [3]. The highest regional morbidity and mortality of this cancer is in Sub-Saharan Africa in which 70,000 new cases every year are reported [4]. Ethiopia as part of the sub-Saharan region shares the regional high incidence and mortality of cervical cancer which makes it one of the major public health problems in the country. It ranks second in cancer-related mortality next to breast cancer among all women and third cause of cancer deaths among reproductive-age women [5]. Annually, an estimated 6,300 new cervical cancer cases are diagnosed and 4884 deaths occur in Ethiopia [6]. Despite, it is a preventable form of cancer both the incidence and mortality figures in national as well as global contexts reflect it as a major reproductive health problem that affects the quality of life of women [7].

One effective strategy for prevention of maternal death that can be resulted from cervical cancer is early detection of the disease and followed by treatment [8]. Even if cervical cancer screening has been the focus of governmental and non-governmental organizations its implementation on the actual ground is limited in which only 12.87% of eligible women utilized the screening service in sub-Saharan Africa [9]. In Ethiopia, the proportion of eligible women who utilized cervical cancer screening services is not much higher than the regional (sub-Saharan Africa) pooled in which only 14.79% of the eligible women were screened [10]. This very low level of early cervical cancer screening leads to an increment in the number of women that presented with full clinical manifestation and advanced stage of the disease that exposed them to poor prognosis and shortest survival. Thus, the global meta-analysis on the proportion of advanced-stage cervical cancer at diagnosis showed that 60.66% of the cervical cancer cases presented at an advanced or late stage (Stage III and IV) of the disease [11].

A late-stage cervical cancer diagnosis is associated with complicated clinical presentation which can result in short survival time and increased mortality [12]. Several factors contribute to the late-stage presentation of cervical cancer patients. Low level of awareness about the disease and its screening, health system-related delays resulting from lack of screening services and diagnostic facilities, and consideration of traditional healing were the most frequently linked factors to advanced disease presentation of cervical cancer patients [13, 14]. Both the cause and consequences of advanced or end-stage cervical cancer were more aggravated in developing countries specifically in Africa, particularly in the sub-Saharan countries [15]. Thus, the largest proportion of cervical cancer patients present with end-stage of disease with poor prognosis and shortest survival in the continent [16].

So far, in Ethiopia, different studies have been conducted and found a highly variable level of late-stage cervical cancer diagnosis and identified different factors that associate with a late presentation in different parts of the country. Even if there was a global systematic review and meta-analysis on late stage cervical cancer diagnosis [11] it is a global pooled which mixes the developing and developed nation (which have advanced diagnostic and treatment modalities) that has great variation in the prevalence as well as on its predictors. Additionally country (nation) specific evidence on the prevalence and predictors of advanced stage cervical cancer diagnosis is necessary for formulation and implementation of focused policy and programs to alleviate the problem. Up to the level of our knowledge, there is no systematic review and meta-analysis done in Ethiopia which can provide summarized evidence on the level of late-stage cervical cancer diagnosis and its determinants; despite the need to summarize the issue and intervene accordingly. Thus, this systematic review and meta-analysis aimed to assess the pooled prevalence of late-stage breast cancer diagnosis and identify its associated factors in Ethiopia.

The evidence from this systematic review and meta-analysis can be used for planning and implementation of an intervention to reduce late-stage cervical cancer presentation in the country. It identifies the important factors that are associated with late-stage cervical cancer diagnosis, it enables respective stakeholders to target and design evidence-based interventions. This study can also serve as a baseline comparison since there is no systematic review and meta-analysis done on this topic. In addition, it may ignite new insight for further studies that might be conducted on related topics.

## Method

### Study design and setting

A systematic review and meta-analysis were conducted on determinants of late-stage cervical cancer diagnosis in Ethiopia. Preferred Reporting Items for Systematic Review and Meta-Analysis guidelines were followed (Supplementary Table 1). PRISMA is a protocol consisting of checklists that guide the conduct and reporting of systematic reviews and meta-analyses, which increase the transparency and accuracy of reviews in medicine and other fields [17]. Ethiopia is one of the low-income countries located in the Horn of Africa with a 2022 projected population of 123.4 million, 133.5 million in 2032, and 171.8 million in 2050 [18]. For administrative purposes, Ethiopia is divided into 11 regions and 2 city administrations. Regions are further classified into zones, and zones are divided into districts. Finally, districts are divided into kebele (the smallest administrative division contains 2000 up to 3500 residents).

### Search strategies and sources of information

We have checked the PROSPERO database (http://www.library.ucsf.edu/) whether published or ongoing projects exist related to the topic to avoid any further duplication. Thus, the findings revealed that there were no ongoing or published articles in the area of this topic. Then this systematic review and meta-analysis were registered in the PROSPERO database with Id no CRD42023441398. Comprehensive literature was searched using international databases PubMed, Embase, Google Scholar, and African Online Journal to retrieve related articles from Jun 1 to 30, 2023. Search terms were formulated using PICO guidelines through online databases. Medical Subject Headings (MeSH) and key terms had been developed using different Boolean operators 'AND' and 'OR'. The following search term was used: "late-stage diagnosis" OR "delayed presentation" OR "delayed diagnosis" OR "advanced stage diagnosis" OR "advanced stage presentation" OR "end-stage diagnosis" OR "end-stage diagnosis" AND “cervical cancer” OR “cervical malignancy” OR “cervical tumor" AND Ethiopia. Grey literature was also searched using Google by exploring the research repository of several universities in the country through their online address using the study topic as a search term.

### Eligibility criteria

This systematic review and meta-analysis included all types of studies conducted in Ethiopia which report the prevalence and determinants of late-stage cervical cancer presentation in the English language, without restriction on race or publication date (until the last search date June 30, 2023). Articles without full abstracts or texts and articles reported out of the outcome interest were excluded. Reviews, meta-analyses, editorials, newspaper articles, and other forms of popular media reports were excluded at each respective stage of screening.

### Outcome measurements

This study has two main outcomes. The primary outcome was the prevalence of late-stage cervical cancer diagnosis. It is defined as the proportion of participants who have presented or were diagnosed in late or advanced stages (stage III to IV) of cervical cancer at first diagnosis. Therefore, all included studies assessed the study participants' stage of disease and categorized them as diagnosed at the late or early stage of the disease. Then, the response was analyzed and presented as the prevalence of late-stage breast cancer diagnosis. The secondary outcome was determinants of late-stage cervical cancer diagnosis.

### Data extraction

All studies obtained from the considered databases were exported to Endnote version X8 software to remove duplicate studies. Then after, all studies were exported to a Microsoft Excel spreadsheet. All authors independently extracted the important data using a standardized data extraction form which was adapted from the Joanna Briggs Institute (JBI) data extraction format. For the first outcome (prevalence) the data extraction format included (primary author, year of publication, regions, study area, sample size, and prevalence with 95% CI) [19]. We extracted data for the second outcome (associated factors to the late-stage cervical cancer diagnosis) using a 2 by 2 table format.

### Quality assessment

To assess the quality of each study included in this systematic review and meta-analysis, the modified Newcastle Ottawa Quality Assessment Scale for cross-sectional studies was used [20] (Supplementary Table 2). Two authors (AZ, AWK) assessed the quality of each study (i.e. methodological quality, sample selection, sample size, comparability and the outcome, and statistical analysis of the study). In the case of disagreement between two authors; another two authors (SS, NK) were involved and discussed and resolved the disagreement.

### Data processing and analysis

The extracted Microsoft Excel spreadsheet format data was imported to STATA version 17 for analysis. Then weighted inverse variance random effect model was used to estimate the pooled prevalence of late-stage cervical cancer presentation in Ethiopia. A forest plot format was used to present the pooled prevalence of late-stage cervical cancer diagnosis with 95%CI [19]. To identify determinants of late-stage cervical cancer diagnosis, a log odds ratio for each factor was calculated.

### Heterogeneity test and publication bias

Cochrane Q-test and I^2^ statistics were computed to assess heterogeneity among all studies. Accordingly, if the result of I^2^ is 0% to 40% it is mild heterogeneity, 40 to 70% would be moderate heterogeneity, and 70 to 100% would be considerable heterogeneity [21]. Funnel plot and Eggers test were done to assess publication bias. The *p*-value > 0.05 indicated that there was no publication bias.

## Result

**Fig. 1:**
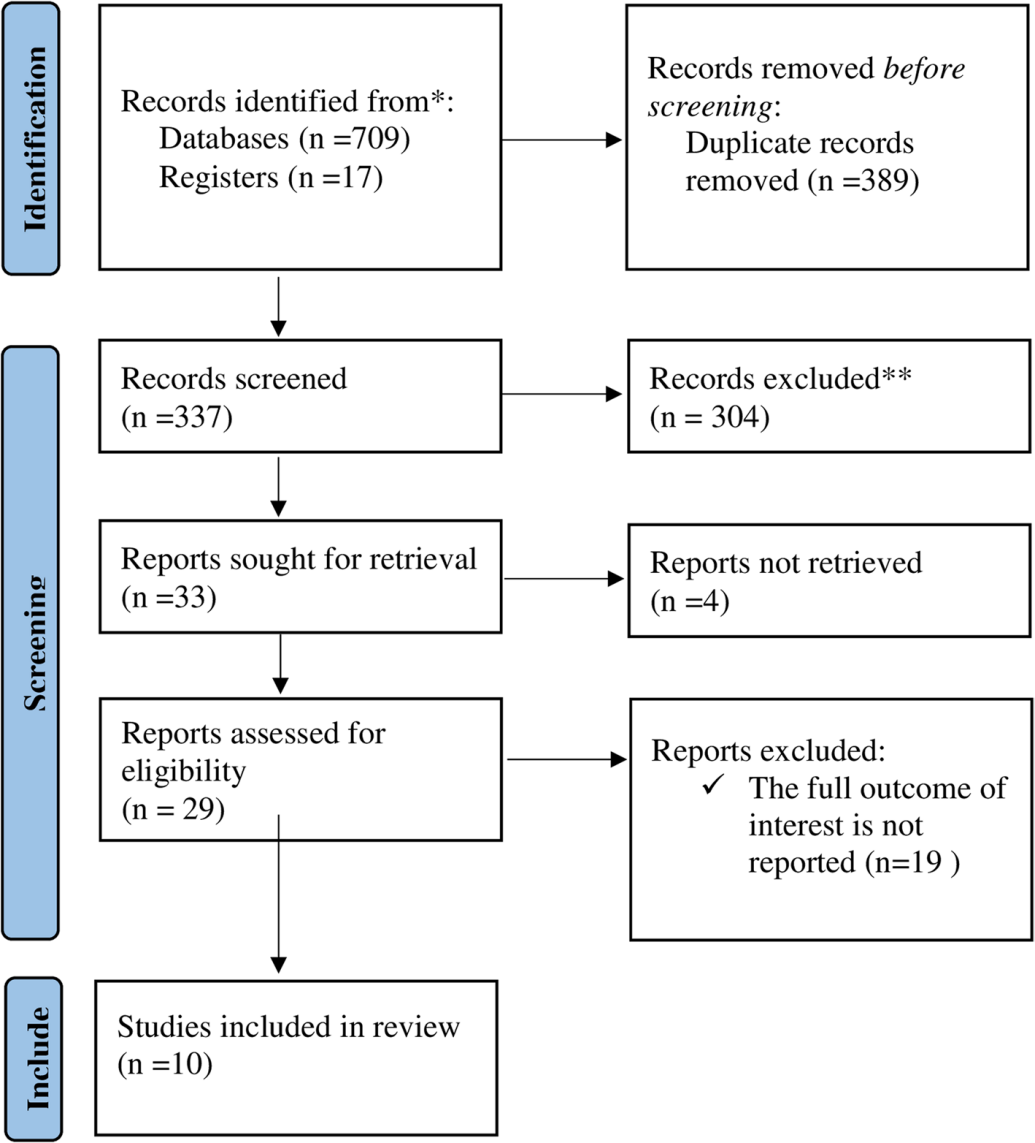
Flow chart of study selection for systematic review and meta-analysis on late-stage cervical cancer diagnosis and its determinant in Ethiopia, 2023

Using our search method, 726 articles were found in the African Journals Online, Embase, Google Scholar, and PubMed databases. 389 duplicate papers were eliminated, 337 items remained. Then, 248 and 56 items were removed after careful examination of their titles and abstracts. Following the evaluation of 33 full-text papers for fulfilment of the inclusion citeria, 23 further articles were excluded for the aforementioned reason. Thus, 10 studies are included in the final systematic review and meta-analysis (Fig. 1).

The included studies in this SRMA comprise 5323 cervical cancer patients. All included studies were cross-sectional. Those studies found a 49.7% to 86.3% prevalence of late-stage cervical cancer diagnosis. Regarding the quality of included studies, the Newcastle Ottawa Quality Assessment scale score of all included studies lies from 7 to 9 which is good (Table 1).

**Table 1:** Characteristics of included studies in the systematic review and meta-analysis on determinants of late-stage cervical cancer diagnosis in Ethiopia

S.no	Author	Period	Study area	Study design	Sample	Prevalence (%)	Study quality
1	Wassie, et al. [22]	2019	Addis Ababa	Crossect	1057	56.8	Good
2	Dereje, et al. [23]	2017- 2018	Addis Ababa	Crossect	212	60.4	Good
3	Zeleke, et al. [24]	2019	Addis Ababa	Crossect	410	86.3	Good
4	Begoihn, et al. [6]	2008 -2012	Addis Ababa	Crossect	1575	56.3	Good
5	Deressa, et al. [25]	2014	Addis Ababa	Crossect	242	66.1	Good
6	Seifu, et al. [26]	2014 – 2019	Addis Ababa	Crossect	346	49.7	Good
7	Solomon, et al. [27]	2010- 2014	Addis Ababa	Crossect	264	52.7	Good
8	Mebratie, et al. [28]	2017 – 2021	Amhara	Crossect	422	63.5	Good
9	Fitiwe W. [29]	2020	Addis Ababa	Crossect	392	53.8	Good
10	Mose O. [30]	2012 – 2014	Addis Ababa	Crossect	403	58.3	Good

### The magnitude of late-stage cervical cancer diagnosis in Ethiopia

**Fig. 2:**
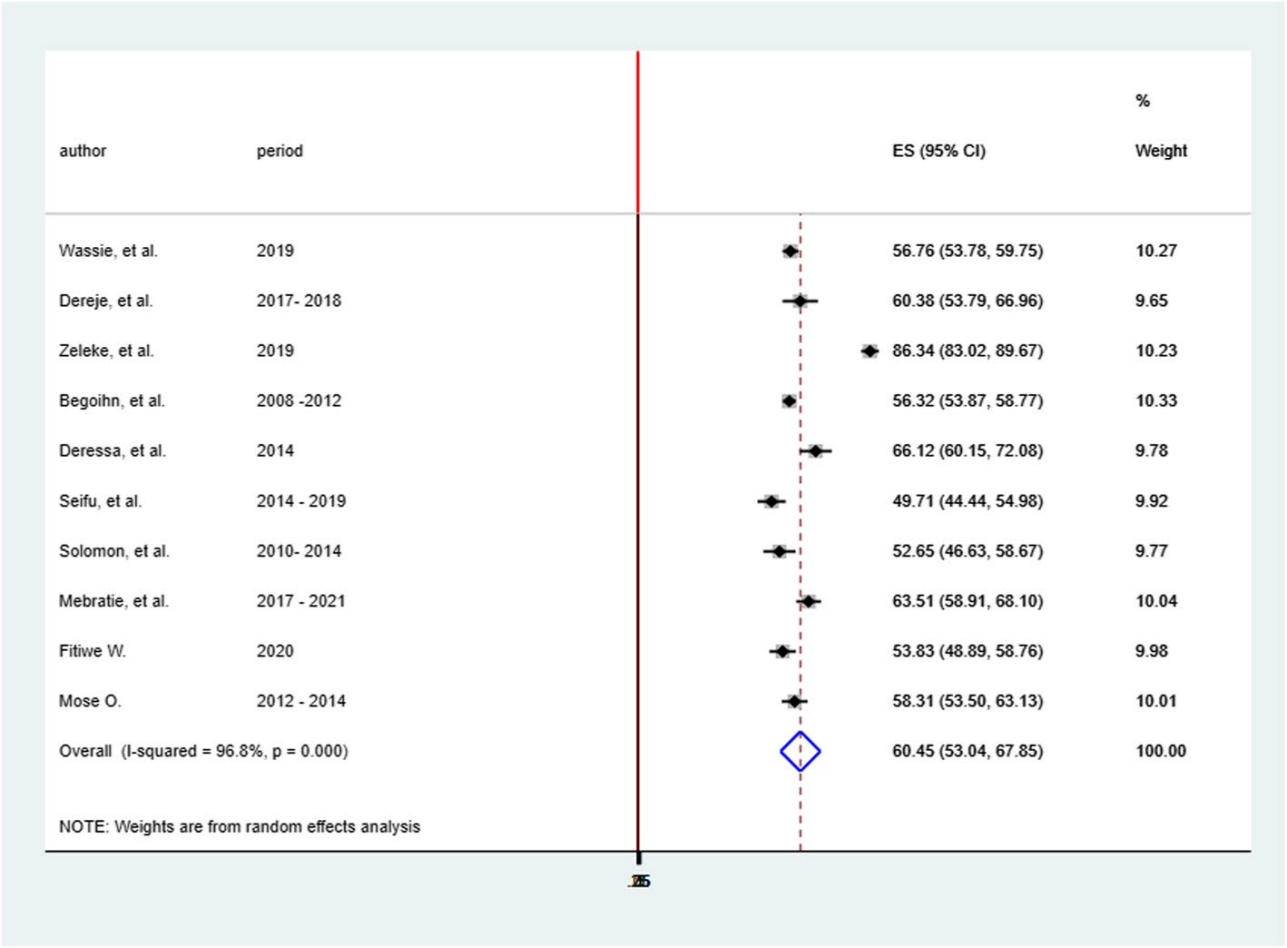
Forest plot showing the Pooled prevalence of late-stage cervical cancer diagnosis in Ethiopia, 2023

The overall late-stage cervical cancer diagnosis in Ethiopia was 60.45% (95%CI; 53.04%-67.85%). The Cochrane heterogeneity index (I^2^ = 96.8%), *P* = 0.000, showed significant heterogeneity of several studies (I^2^ > 70%). A forest plot was used to display the results (Fig. 2).

### Publication bias

**Fig. 3:**
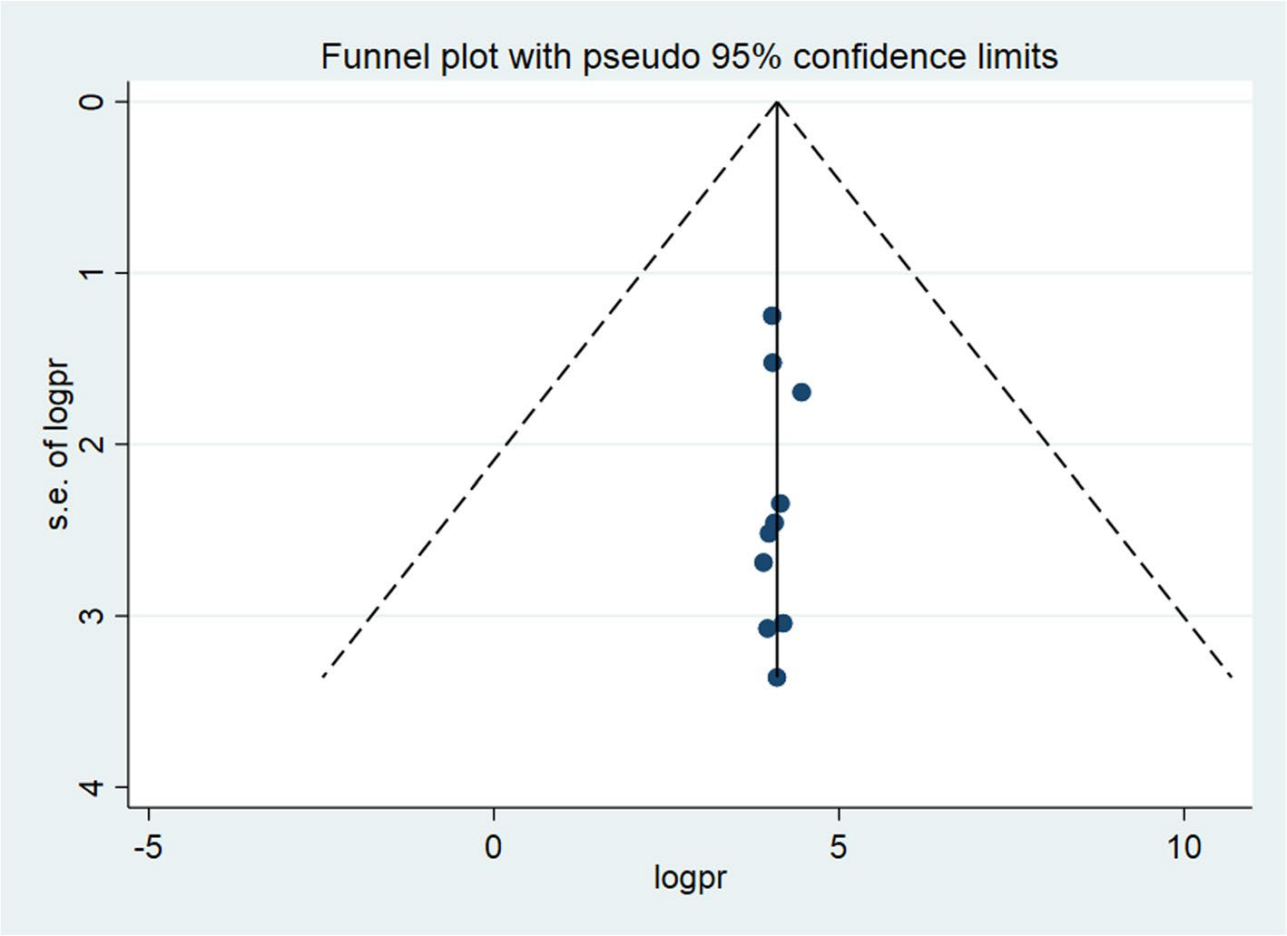
Funnel plot showing the symmetric distribution of articles on late-stage cervical cancer diagnosis and its determinant in Ethiopia, 2023

A funnel plot was used in this systematic review and meta-analysis to test for publication bias at a significance level lower than 0.05. The funnel plot (Fig. 3) and Egger's regression test's *P* = 0.793 (*p* > 0.05) (statistically not significance) indicated that there was no evidence of publication bias.

### Sensitivity analysis

**Fig. 4:**
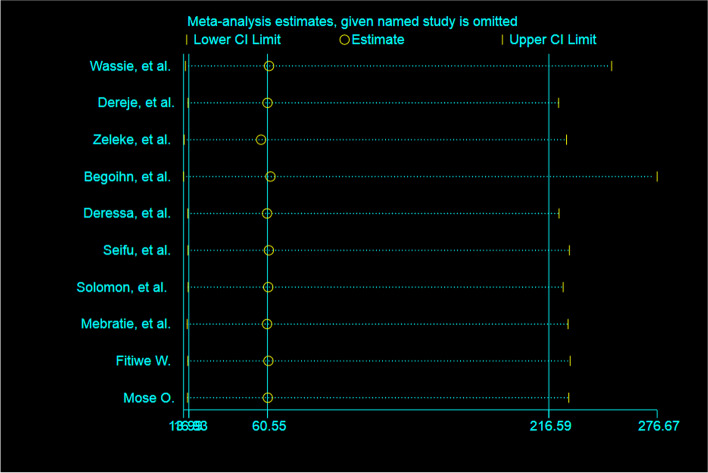
Sensitivity analysis of late-stage cervical cancer diagnosis in Ethiopia, 2023

No single study had affected the overall pooled prevalence of late-stage cervical cancer detection, according to a random-effects model result (Fig. 4).

### Determinants of late-stage cervical cancer diagnosis in Ethiopia

The identification of the determinants of late-stage cervical cancer diagnosis in our review includes three variables that are associated to late-stage cervical cancer diagnosis in two or more primary studies. As a result, in Ethiopia, late-stage cervical cancer detection was associated with poor awareness about of cervical cancer and its treatment, patient delay in seeking care, and rural residence. Patients who have poor awareness about cervical cancer and its treatment were 1.55 times more likely to present with late-stage cervical cancer as compared to their counterparts (AOR = 1.55, 95% CI: (1.03 – 2.33)). Similarly, Patients who had a longer delay to seek care were 1.02 times more likely to be diagnosed at an advanced stage as compared to patients who seek care early (AOR = 1.02, 95% CI: (1.01 – 1.03)). Moreover, patients from rural areas were twice more likely to be present at the advanced stage as compared to urban residing patients (AOR = 2.07, 95% CI:( 1.56 – 2.75)) (Table 2).

**Table 2:** Factors associated with late-stage cervical cancer diagnosis in Ethiopia

Variable	Authors	AOR	95%CI	Pooled AOR	95%CI of pooled AOR
Poor awareness	Zeleke, et al	1.33	1.01–2.71	1.55	1.03 – 2.33
	Fitiwe W	2.38	1.03 – 5.11		
Longer patient delay to seek care	Dereje, et al	1.31	1.04– 1.71	1.02	1.01 – 1.03
	Begoihn, et al	1.004	1.002 – 1.006		
Rural residence	Wassie, et al	1.88	1.38 – 2.56	2.07	1.56 – 2.75
	Fitiwe W	3.41	1.69 – 6.88		

## Discussion

Even though cervical cancer is the most preventable form of cancer through human papillomavirus vaccination, effective screening, and treatment of pre-cancerous lesions, it is the most prevalent malignancy among women in developing countries [31]. Due to the poor healthcare setup for early screening and treatment, most patients are diagnosed at an advanced stage and the disease's related mortality is also higher in low and middle-income countries [32]. Globally, also 60.66% of the cervical cancer cases presented at an advanced or late stage (Stage III and IV) of the disease, and the largest share was from Asia (69.3%) and Africa (62.6%) [11]. late-stage diagnosis of cervical cancer resulted in complicated clinical manifestation and poor prognosis which significantly diminish survival [33].

In Ethiopia, several primary studies were conducted and found a variable level of late-stage cervical cancer diagnosis in the country; however, summarized and representative evidence on the level and factors associated with late-stage cervical cancer presentation as a nation was scarce. Thus this SRMA aimed to determine the level and identify contributing factors to late-stage cervical cancer diagnosis in the country. Accordingly, the pooled prevalence of late-stage cervical cancer diagnosis in Ethiopia was 60.45% (95%CI; 53.04%-67.85%). The finding was consistent with study findings from Kenya (53.9%) [34], Uganda (66%) [35], Tanzania (63.9%) [14] Ghana (65.97%) [36], and with the global pooled prevalence of late stage cervical cancer presentation (60.66%) [11]. It was slightly lower than the study finding from Sudan [37] and Nigeria [38]. However, the finding was higher than studies from Morocco (39.9) [39], Mexico (38%) [40], India (39.5%) [41], and England (2.3%) [42]. The possible justification for the existing discrepancy may be the difference in health care setup that the respective country had for early detection of cervical cancer; since the above-listed countries are economically good and have advanced diagnostic facilities that enable them to early detect and treat the disease. Whereas in Ethiopia the infrastructure and the whole health system setup for early detection of cancer are not much pioneering that is why a significant proportion of cervical patients presented with late stage. The finding calls for action to be taken to improve screening of cervical cancer and effort should be made in reducing barriers to early diagnosis of cervical cancer to reduce the proportion of patients that are presented at the end stage of the disease to improve prognosis and survival.

Reducing the proportion of patients presenting with late-stage of cervical cancer requires intervention that targets the contributing factors for late-stage presentation and the barriers to screening. Thus, our review identified poor awareness about cervical cancer and its treatment, patient delays to seek care, and rural residence as contributing factors to late-stage presentation at first diagnosis. Patients who have poor awareness about cervical cancer and its treatment were 55% times more likely to present with late-stage cervical cancer as compared to their counterparts. The finding was in line with study findings in Morocco [39] and Kenya [43] in which patients who have better knowledge about the cause, consequences, and treatment of cervical cancer are less likely to delay for diagnosis and treatment. The finding implies the need to improve public awareness about cervical cancer including its cause, consequence, diagnosis approach, treatment, and prevention methods so as to enable women to be involved in screening and early diagnosis and treatment.

In this review, patients who had a longer delay to seek care were more likely to be diagnosed at an advanced stage as compared to patients who seek care early. The finding was consistent with a study in Nepal in which patients who were late to share their first symptom were prone to advanced-stage diagnosis [44]. This is because patients who visited health institutions early; while they feel the first symptom have a great opportunity to be diagnosed in the early disease phase that can be treated easily and cured. The finding implies the need to expand awareness creation about early symptoms of cervical cancer and improve health-seeking behavior to minimize the delay of patients in seeking cervical cancer diagnosis and treatment care.

Moreover in our review, rural cervical cancer patients were twice more likely to be present at the advanced stage as compared to urban residing patients. This is in line with the global meta-analysis of factors associated with late-stage cervical cancer presentation [11], with studies in Sudan [37] and Morocco [45] in which rurally residing cervical cancer patients are more frequently presented at the late stage of the disease. This might be due to the infrastructure in the rural environment including the health care setup may expose them to both patient and health-system delays which leads them to late-stage presentation. Additionally, patients who live in rural areas cannot also get adequate information about cervical cancer which leads them delayed diagnosis [46]. The finding suggests that cervical cancer screening and awareness creation intervention should target the rurally residing segments of the population to lower the proportion of cervical cancer victims who are presented at the late stage of the disease.

Not standing with its finding, this SRMA has limitation. Our search strategy found primary studies of low methodological quality which are all cross-sectional, especially we have not got high quality randomized control trial studies to increase the validity of the finding.

## Conclusion

Six of every ten cervical cancer cases in Ethiopia were diagnosed at an advanced stage, which indicates the health system managers in the country should carefully involve in planning and implementation interventions to lower the proportion of patients diagnosed at the end stage of the disease to enhance prognosis and survival using this current and representative evidence as base line. Poor awareness about cervical cancer and its treatment, long patient delay to seek care, and rural residence were positively associated with late–stage diagnosis. Therefore intervention efforts at all level of the health system setup should be made in improving public awareness about the cause, consequences, diagnosis, and treatment and prevention approach of cervical cancer. Minimizing patient delay to seek care and expansion of screening services specifically in the rural residing segment of the population should be the focus of intervention so as to detect the disease early and improve survival. Future researches should focus in exploring the determinants of late stage cervical cancer diagnosis using high quality study methods.

### Supplementary Information

**Additional file 1:** **Supplementary table 1. **PRISMA 2020 Checklist followed for this systematic review and meta-analysis.

**Additional file 2:** **Supplementary table 2. **Newcastle-Ottawa Quality Assessment Scale for cross sectional studies used in the systematic review and meta-analysis 2023.

## Data Availability

All data generated or analyzed during this study are included in this published article and its supplementary information files.

## Abbreviations

PRISMA: Preferred Reporting Items for Systematic Reviews and Meta-Analyses
SRMA: Systematic Reviews and Meta-Analyses
