# Supplementary material for: Determinants of late-stage cervical cancer presentation in Ethiopia: a systematic review and meta-analysis
Source: BMC Cancer. 2023 Dec 14;23:1228. doi: 10.1186/s12885-023-11728-y (PMC10720221; doi:10.1186/s12885-023-11728-y)
Supplement: Supplementary file 2 — Additional file 2: Supplementary table 2. Newcastle-Ottawa Quality Assessment Scale for cross sectional studies used in the systematic review and meta-analysis 2023. [file 12885_2023_11728_MOESM2_ESM.docx]

**Supplementary table 2:** Newcastle-Ottawa Quality Assessment Scale for cross sectional studies used in the systematic review and meta-analysis 2023

|  | Selection | | | | Comparability | Outcome | | Total score |
| --- | --- | --- | --- | --- | --- | --- | --- | --- |
| Authors | Representativeness (1) | Sample size (1) | Non respondents (1) | Ascertainment of the exposure (risk factor) (2) | The subjects in different outcome groups are comparable (1) | Assessment of the outcome (2) | Statistical test (1) |  |
| Wassie, et al. | 1 | 1 | 1 | 2 | 1 | 2 | 1 | 9 |
| Dereje, et al. | 1 | 0 | 1 | 2 | 1 | 2 | 1 | 8 |
| Zeleke, et al. | 1 | 1 | 1 | 2 | 1 | 1 | 1 | 8 |
| Begoihn, et al. | 1 | 1 | 1 | 2 | 1 | 2 | 1 | 9 |
| Deressa, et al. | 1 | 0 | 1 | 2 | 1 | 2 | 0 | 7 |
| Seifu, et al. | 1 | 1 | 1 | 2 | 1 | 1 | 1 | 8 |
| Solomon, et al. | 1 | 1 | 1 | 1 | 1 | 2 | 1 | 8 |
| Mebratie, et al. | 1 | 1 | 1 | 1 | 1 | 2 | 1 | 8 |
| Fitiwe W. | 1 | 1 | 1 | 2 | 1 | 1 | 1 | 8 |
| Mose O. | 1 | 1 | 1 | 2 | 1 | 1 | 1 | 8 |
